# Taste Perception: Cracking the Code

**DOI:** 10.1371/journal.pbio.0020064

**Published:** 2004-03-16

**Authors:** Jane Bradbury

## Abstract

Our sense of taste begins with taste buds and ends in the brain. Researchers are beginning to unravel the mechanisms and connections that lie in between

The ability to taste food is a life-and-death matter. Failure to recognise food with a high enough caloric content could mean a slow death from malnutrition. Failure to detect a poison could result in near-instant expiration. And now, as researchers begin to understand some of the nuts and bolts of taste perception, it seems that the sense of taste may also have more subtle effects on health.

## The Basics of Taste

**Figure 1:**
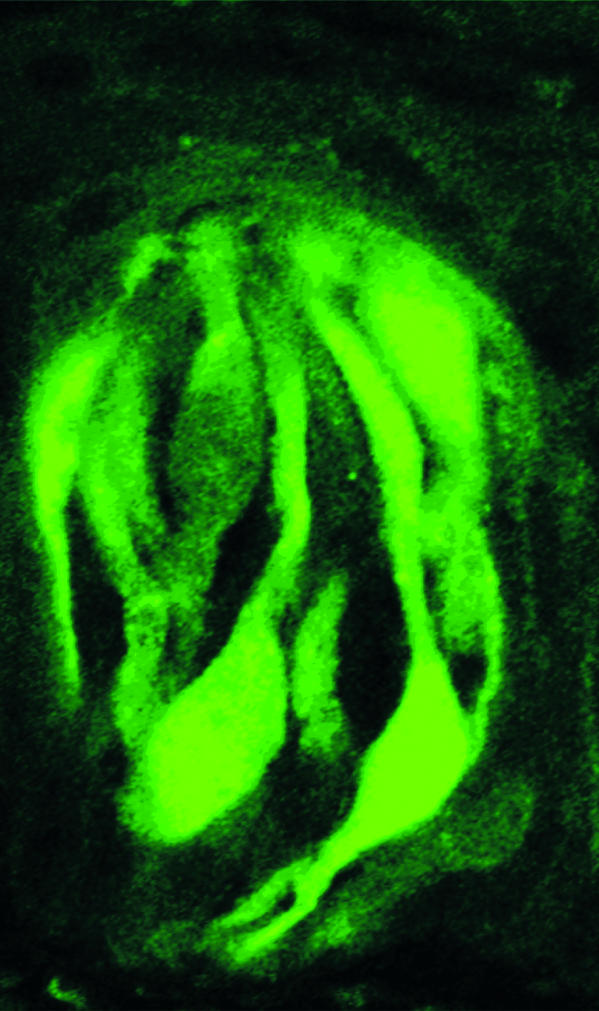
A Taste Bud in a Mouse This taste bud was taken from a transgenic mouse in which the marker green fluorescent protein is being driven by the T1R3 promoter; 20%–30% of the cells in the taste bud are expressing T1R3. (Photograph courtesy of Sami Damak, Mount Sinai School of Medicine, New York, New York, United States.)

At the front line of the taste sensory system are the taste buds—onion-shaped structures on the tongue and elsewhere in the mouth (Figure 1). Up to 100 taste receptor cells—epithelial cells with some neuronal properties—are arranged in each taste bud. In the tongue, the taste buds are innervated by the chorda tympani (a branch of the facial nerve) and the glossopharyngeal nerve. These nerves carry the taste messages to the brain.

Taste is the sense by which the chemical qualities of food in the mouth are distinguished by the brain, based on information provided by the taste buds. Quality or ‘basic taste’, explains Bernd Lindemann, now retired but an active taste researcher in Germany for many years, is a psychophysical term. Large numbers of people describe different tastants and then statistical analyses are used to define the important tastes. ‘The number of taste qualities has varied over the years’, says Lindemann. ‘We are now settling at around five, though I would not be surprised if some additional qualities pop up’.

The five qualities that Lindemann refers to are salty, sour, bitter, sweet, and umami, the last being the Japanese term for a savoury sensation. Salty and sour detection is needed to control salt and acid balance. Bitter detection warns of foods containing poisons—many of the poisonous compounds produced by plants for defence are bitter. The quality sweet provides a guide to calorie-rich foods. And umami (the taste of the amino acid glutamate) may flag up protein-rich foods. Our sense of taste has a simple goal, explains Lindemann: ‘Food is already in the mouth. We just have to decide whether to swallow or spit it out. It's an extremely important decision, but it can be made based on a few taste qualities’.

## From Physiology to Molecular Biology

Taste has been actively researched for many decades. During the 20th century, electrophysiologists and other researchers worked hard to understand this seemingly simple sense system. Then, in 1991, the first olfactory receptors were described. These proteins, which are exposed on the surface of cells in the nose, bind to volatile chemicals and allow us to detect smells. This landmark discovery, in part, encouraged many established taste researchers to investigate the molecular aspects of taste.

The olfaction results also enticed researchers from other disciplines into the taste field, including collaborators Charles Zuker (University of California, San Diego [UCSD], La Jolla, California, United States) and Nick Ryba (National Institute of Dental and Craniofacial Research [NIDCR], Bethesda, Maryland, United States). About six years ago, explains Zuker, who previously worked on other sensory systems in flies, ‘there was a disconnect between our understanding of sensations in the case of photoreception, mechanoreception, touch, and so on and what we knew about taste’. There was evidence, says Ryba, that a class of protein receptors called G-protein-coupled receptors (GPCRs) were involved in sweet and bitter taste, ‘but the receptors weren't known, so we started to look for them …. These molecules are intrinsically interesting, but more importantly, they provide tools with which we can dissect out how taste works’.

## Bitter, Sweet, and Umami Receptors

The bitter receptors fell first to the onslaught of the UCSD–NIDCR team and other molecular biologists. In 1999, the ability to taste propylthiouracil, a bitter tasting compound, had been linked to a locus on human Chromosome 5p15. Reasoning that this variation might be due to alterations in the coding sequence for a bitter receptor, the UCSD–NIDCR researchers used the draft of the human genome to search for sequences that resembled GPCRs on Chromosome 5p15. ‘That was how we found T2R1, the first bitter receptor, and, subsequently, a whole family of T2Rs’, says Zuker.

*Researchers want to know: how is taste coded?*

All these receptors, says Zuker, are coexpressed in bitter taste receptor cells, a result that contradicts other research showing that different bitter-responsive cells react to different bitter molecules. ‘To me’, says Zuker, ‘it makes sense that all the bitter receptors would be expressed in each bitter taste cell. We just need to know if something is bitter to avoid death’, not the exact identity of the bitter tastant.

The sweet receptor story started in 1999 with the identification of two putative mammalian taste receptors, GPCRs now known as T1R1 and T1R2. In early 2001, four groups reported an association between the mouse *Sac* locus, which determines the ability of mice to detect saccharin, and T1R3, a third member of the T1R family. The UCSD–NIDCR team subsequently showed that the T1R2 and T1R3 heterodimer (a complex of one T1R2 and one T1R3 molecule) forms a broadly tuned sweet receptor, responsive to natural sugars and artificial sweeteners, and that a homodimer of two T1R3 molecules forms a low-affinity sugar receptor that responds to high concentrations of natural sugars only. All sweet detection, concludes Zuker, is via the T1R2 and T1R3 receptors.

And umami? A truncated glutamate receptor was identified as an umami receptor by researchers at the University of Miami (Florida, United States) School of Medicine in 2000. Zuker, however, believes that the one and only umami receptor is a heterodimer of T1R1 and T1R3. In October 2003, Zuker and his coworkers reported that mice in which either T1R1 or T1R3 has been knocked out show no preference for monosodium glutamate (MSG), an umami tastant. However, other researchers reported in August 2003 that T1R3 knockouts retain some preference for MSG. ‘We believe this is due either to the truncated glutamate receptor or another unknown receptor’, says lead author Sami Damak (Mount Sinai School of Medicine, New York, New York, United States). Damak says he does not know why the two sets of T1R3 knockout mice behaved differently, but the UCSD–NIDCR researchers suggest that the residual response to MSG seen by Damak et al. is a response to the sodium content of MSG. Damak is not alone, however, in thinking there may be more than one umami receptor (and additional sweet receptors).

Commenting on these recent discoveries, taste expert Linda Bartoshuk (Yale University School of Medicine, New Haven, Connecticut, United States) says that ‘it is lovely to see all these details, especially as they confirm what we already believed conceptually’. For example, she says, it is no surprise that there are many bitter receptors but probably only one sweet receptor. ‘There are so many poisons and it makes perfect sense to have lots of receptors feeding into a common transduction pathway. Sweet is a different problem. In nature, there are many molecules with structures similar to sugar that we must not eat because we cannot metabolise them. So I would have predicted one or at most a few highly specific sweet receptors’.

## What about Salty and Sour Receptors?

The salty and sour receptors may be very different from the GPCRs involved in bitter, sweet, and umami perception, which bind complex molecules on the outside of the cell and transmit a signal into the cell. For salty and sour perception, the taste cell only needs to detect simple ions. One way to do this may be to use ion channels—proteins that form a channel through which specific inorganic ions can diffuse. Changes in cellular ion concentrations could then be detected and transmitted to the nervous system.

Physiologist John DeSimone (Virginia Commonwealth University, Richmond, Virginia, United States) says there are at least two ion channel receptors for salt in rodent taste receptor cells. The first of these is the epithelial sodium channel, a widely expressed channel that can be blocked specifically with the drug amiloride. In rats, says DeSimone, only 75% of the nerve response to salt can be blocked by amiloride, so there is probably a second receptor. This, he says, seems to be a generalist salt receptor—the amiloride-sensitive channel only responds to sodium chloride—and may be the more important receptor in people.

Sour tastants are acids, often found in spoiled or unripe food. DeSimone's current idea is that strong acids enter taste cells through a proton channel (probably a known channel present on other cell types) while weak acids, like acetic acid (vinegar), enter as neutral molecules and then dissociate to lower intracellular pH. DeSimone believes that he has identified the proton channel involved in sour taste as well as an ion channel that could be the second salt receptor, and he plans to do knockout experiments on both. If these channels are essential elsewhere in the body, as DeSimone suspects, to avoid lethality he will need to construct conditional knockouts in which the channel is lost only in the taste receptor cells.

Zuker, meanwhile, is not convinced that the current ion channel candidates for salt and sour perception are correct. And, he says, GPCRs could also be involved in these modalities. ‘There is a precedent for that’, he claims, noting that extracellular calcium is sensed by a GPCR.

## Taste-Coding

With many taste receptors now identified, researchers are turning to a long-standing question in taste perception: how is taste coded? When we eat, our tongue is bombarded with tastants. How is their detection and transduction of information organised so that the appropriate response is elicited? Taste physiologist Sue Kinnamon (Colorado State University, Fort Collins, Colorado, United States) explains the two theories of taste-coding. In the ‘labelled-line’ model, sweet-sensitive cells, for example, are hooked up to sweet-sensitive nerve fibres that go to the brain and code sweet. If you stimulate that pathway, says Kinnamon, ‘you should elicit the appropriate behavioural response without any input from other cell types’. In the ‘cross-fibre’ model, the pattern of activity over many receptors codes taste. This model predicts that taste receptor cells are broadly tuned, responding to many tastants. Support for this theory, says Kinnamon, comes from electrical recordings from receptor cells and from nerves innervating the taste buds that show that one cell can respond to more than one taste quality.

Zuker and Ryba's recent work strongly suggests that taste-coding for bitter, sweet, and umami fits the labelled-line model in the periphery of the taste system. Their expression data show that receptors for these qualities are expressed in distinct populations of taste cells. In addition, in early 2003, they reported that, as in other sensory systems, a single signalling pathway involving the ion channel TRPM5 and PLCβ2, a phospholipase that produces a TRPM5 activator, lies downstream of the bitter, sweet, and umami receptors. When the UCSD–NIDCR researchers took PLCβ2 knockout mice, which did not respond to bitter, sweet, or umami, and engineered them so that PLCβ2 was only expressed in bitter receptor-expressing cells, only the ability to respond to bitter tastants was regained. These data, says Zuker, support the labelled-line model.

The latest data supporting the labelled-line model came last October when Zuker and colleagues described mice in which a non-taste receptor—a modified κ-opioid receptor that can only be activated by a synthetic ligand—was expressed only in cells expressing T1R2, sweet-responsive cells. The mice were attracted to the synthetic ligand, which they normally ignore, indicating that dedicated pathways mediate attractive behaviours. The researchers plan similar experiments to see whether the same is true for aversive behaviours.

Even with all these molecular data, the cross-fibre model of taste-coding still has its supporters—just how many depends on whom one talks to. Both Damak and Kinnamon, for example, believe that there is at least some involvement of cross-fibre patterning even in the taste receptor cells. But, says neurobiologist and olfaction expert Lawrence C. Katz (Duke University, Durham, North Carolina, United States), ‘the onus is now on people who believe otherwise [than the labelled-line model] to provide compelling proof for the cross-fibre theory because now, at least at the periphery, the evidence is compelling for a labelled line for bitter, sweet, and umami’. Bartoshuk also says the debate is decided in favour of the labelled-line model in the periphery. The crossfibre model is an interesting historical footnote, she comments.

Whether this putative link between taste perception and health can be confirmed and whether it will be possible to manipulate food preferences to improve health remain to be seen. However, it seems certain that, as in the past five years, the next five years will see large advances in our knowledge of many aspects of taste, a fascinating and important sensory system.

## What Next—and Why Study Taste Anyway?

The periphery of the taste sensory system has yielded many of its secrets, but relatively little is known about the transduction pathways in taste, how taste cells talk to the nervous system, or about events further downstream in the brain. How are signals from taste receptors integrated with those from olfactory receptors to form a representation of complex food flavours, for example? With their expanding molecular toolbox, researchers can now delve deeper into these aspects of taste perception. This may tell us not only about taste but about how the nervous system in general is put together, says Ryba.

But understanding taste is not just an academic exercise. It has practical uses too. DeSimone suggests that by understanding salt receptors, it may be possible to design artificial ligands to help people lower their salt intake. As Kinnamon succinctly puts it, ‘Can you imagine eating potato chips and not having the salty component?’ An artificial salt receptor ligand could make salt-free foods a palatable option for people with high blood pressure. Lindemann also sees a great future in artificial ligands for taste receptors. The sense of taste is partly lost in elderly people, he says, so better tastants—effectively ‘chemical spectacles’—might give them back their pleasure of eating and thereby improve their quality of life.

**Figure 2:**
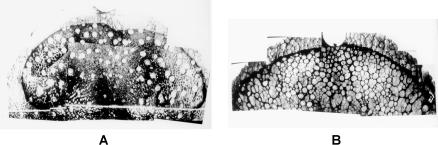
Non-Taster or Supertaster? (A) Top surface of the tongue of a non-taster. (B) Tongue of a supertaster. The small circles are fungiform papillae, each of which contains about six taste buds.

Finally, some aspects of taste may be inextricably tied up with general health, says Bartoshuk. Many people who can taste propylthiouracil are also ‘supertasters’—they have more fungiform papillae, structures containing taste buds, on their tongues than non-tasters (Figure 2). Supertasters find vegetables bitter—particularly brassicas, like Brussel sprouts—so they tend to eat fewer vegetables as part of their regular diet than non-tasters. ‘Being a supertaster affects your taste preferences, your diet, and ultimately your health’, claims Bartoshuk.

## Abbreviations

GPCR: G-protein-coupled receptor
MSG: monosodium glutamate
